# The association of proBNPage with manifestations of age-related cardiovascular, physical, and psychological impairment in community-dwelling older adults

**DOI:** 10.1007/s11357-021-00381-7

**Published:** 2021-05-13

**Authors:** Antonio Muscari, Giampaolo Bianchi, Paola Forti, Donatella Magalotti, Paolo Pandolfi, Marco Zoli, the Pianoro Study Group

**Affiliations:** 1 Department of Medical and Surgical Sciences, University of Bologna, Via Albertoni, 15 40138 Bologna, Italy; 2 Medical Department of Continuity of Care and Disability, IRCCS Azienda Ospedaliero-Universitaria di Bologna, Bologna, Italy; 3 Epidemiological and Health Promotion Unit, Department of Public Health, AUSL Bologna, Bologna, Italy

**Keywords:** Aging, Biological age, Epidemiological studies, NT-proBNP

## Abstract

NT-proB-type natriuretic peptide (NT-proBNP) serum concentration can be transformed by simple formulas into proBNPage, a surrogate of biological age strongly associated with chronological age, all-cause mortality, and disease count. This cross-sectional study aimed to assess whether proBNPage is also associated with other manifestations of the aging process in comparison with other variables. The study included 1117 noninstitutionalized older adults (73.1 ± 5.6 years, 537 men). Baseline measurements of serum NT-proBNP, erythrocyte sedimentation rate, hemoglobin, lymphocytes, and creatinine, which have previously been shown to be highly associated with both age and all-cause mortality, were performed. These variables were compared between subjects with and without manifestations of cardiovascular impairment (myocardial infarction (MI), stroke, peripheral artery disease (PAD), arterial revascularizations (AR)), physical impairment (long step test duration (LSTD), walking problems, falls, deficit in one or more activities of daily living), and psychological impairment (poor self-rating of health (PSRH), anxiety/depression, Mini Mental State Examination (MMSE) score < 24). ProBNPage (years) was independently associated (OR, 95% CI) with MI (1.08, 1.07–1.10), stroke (1.02, 1.00–1.05), PAD (1.04, 1.01–1.06), AR (1.06, 1.04–1.08), LSTD (1.03, 1.02–1.04), walking problems (1.02, 1.01–1.03), and PSRH (1.02, 1.01–1.02). For 5 of these 7 associations, the relationship was stronger than that of chronological age. In addition, proBNPage was univariately associated with MMSE score < 24, anxiety/depression, and falls. None of the other variables provided comparable performances. Thus, in addition to the known associations with mortality and disease count, proBNPage is also associated with cardiovascular manifestations as well as noncardiovascular manifestations of the aging process.

## Introduction

In recent years, an increasing number of studies have been performed to search for markers or groupings of markers capable of providing an estimate of biological age (e.g., a reliable estimate independent of chronological age of how close to death each individual is) [1–12]. In addition to its evident prognostic use, this information could prove particularly useful as a surrogate endpoint in place of mortality in the search for anti-aging therapies [13]. One or 2 years of follow-up could be sufficient to demonstrate the ability of a therapy to slow down or revert the increase in biological age instead of decade-long studies to assess differences in mortality between a treated and a placebo group.

Keeping in mind these objectives, any candidate marker of biological age should at least be correlated with chronological age and be able to predict life span better than chronological age [14, 15]. In a recent study [16], we demonstrated that the N-terminal fragment of B-type natriuretic peptide precursor (NT-proBNP), which is commonly utilized for the diagnosis of heart failure and is easily measurable with commercial kits, has both characteristics. A subsequent study by Crimmins et al. [12] confirmed that among 24 possible biomarkers of the aging process, NT-proBNP was the one most correlated with chronological age. The serum concentrations of NT-proBNP in pg/ml differ between the two sexes [17, 18], but with two simple formulas, they can be transformed into a “proBNPage” in years, which is valid for both sexes [16]. ProBNPage is normally distributed, and its mean value is similar to that of the chronological age of the studied population. However, its standard deviation is much wider, reflecting the variability of biological age; in fact, some individuals are “younger” and others are “older” than their chronological age.

In our previous study [16], in addition to NT-proBNP, 10 other laboratory variables were considered that had previously been used as possible markers of biological age [6, 11, 19, 20]. However, in a Cox regression concerning the assessment of all-cause mortality during a follow-up of 7 years, none of these 10 variables was found to be independently associated with mortality. Only proBNPage and chronological age remained associated with mortality, with a slight prevalence of proBNPage over chronological age. In addition to all-cause mortality, proBNPage was associated with disease count, which was in agreement with the fact that an increase in NT-proBNP was also found to be associated with extracardiac pathologies [21] and with mortality in subjects without heart disease [22–24].

Nevertheless, the possible relationships involving proBNPage not only with death and the number of diseases but also with aging-related negative events remain to be examined. In particular, the aging process is often characterized by variable degrees of impairment of physical and mental health [25–27], and B-type natriuretic peptides have been found to be inversely associated with both muscle strength [28–30] and cognitive function and are even able to predict the risk of dementia [31–34].

Thus, utilizing the same sample of an older adult population of the previous study, we performed the present cross-sectional investigation with the objective of seeking the possible associations between proBNPage and a set of cardiac-cerebral-vascular, physical, and mental aspects of aging in comparison with other laboratory variables.

## Methods

### Patients

From November 2003, all 3255 inhabitants of the Pianoro municipality (Northern Italy) aged ≥ 65 years were invited to participate in this study, which was mainly aimed at assessing the cardiovascular effects of physical activity in an older adult population. They were all community-dwelling, noninstitutionalized, nonhospitalized subjects. No other exclusion criteria were adopted in the main analysis. However, patients with atrial fibrillation or a history of heart failure were excluded in a sensitivity analysis (see further). Two thousand twenty-two subjects returned a preliminary postal questionnaire, and of them, 1163 agreed to be subsequently examined in our laboratories from December 2003 to January 2005. The characteristics of the participants as well as of those who did not participate in the second phase have been reported in detail in previous publications [23, 35, 36]. Forty-six subjects were excluded due to missing data, and all the remaining 1117 subjects were utilized for our analyses (age range 65–93 years at the time of enrollment, mean age ± SD, 73.1 ± 5.6 years, 537 men, and 580 women). In contrast to our previous study [16], which assessed the association of NT-proBNP with chronological age, the present study was performed without excluding subjects with possible cardiovascular causes of NT-proBNP increase (particularly atrial fibrillation and heart failure) from the primary analysis. In fact, in this case, the object of the study was the relationship between proBNPage and some manifestations of the aging process, and cardiovascular pathologies may be important determinants of biological age. However, we also verified the main results of this study in a sensitivity analysis after exclusion of subjects with a history of atrial fibrillation or heart failure (*N* = 31).

The study was approved by our joint University-Hospital Ethics Committee, and all participants provided their signed informed consent.

### Laboratory variables associated with aging

In a previous study [16], we assessed 11 laboratory variables: blood glucose, cholesterol, albumin, hemoglobin, mean cell volume, leukocytes, lymphocytes, creatinine, erythrocyte sedimentation rate (ESR), high-sensitivity C-reactive protein, and NT-proBNP (subsequently transformed into proBNPage). Because any marker of biological aging should be strongly associated with both chronological age and all-cause mortality, in the present study, we considered only the 5 variables that in univariate analysis were associated with age with *P* values < 0.0001 and with mortality with *P* values < 0.005: proBNPage (years), ESR (mm/h), hemoglobin (g/dl), lymphocytes (%), and creatinine (mg/dl).

ProBNPage was calculated in the two sexes with the following formulas [16]: proBNP age men = [log(NT-proBNP) + 1.2068]/0.0827 and proBNP age women = [log(NT-proBNP) − 1.5258]/0.0478.

Venous blood samplings were drawn in the morning after a 12-h fast. All measurements were performed using commercially available kits. In particular, serum levels of NT-proBNP were measured by an electrochemiluminescence immunoassay (proBNP Elecsys, Roche Diagnostics, Mannheim, Germany), the complete blood count was obtained by an automated counter (Bayer ADVIA 120), and the erythrocyte sedimentation rate (ESR) was automatically measured by the stopped-flow technique in a capillary microphotometer (Alifax Test 1 System).

### Manifestations of aging

The manifestations of aging considered in this study were grouped into 3 categories: cardiac-cerebral-vascular events, deficits of physical function, and psychological or mental complaints.

The cardiac-cerebral-vascular events included previous myocardial infarction, previous ischemic or hemorrhagic stroke, peripheral artery disease, and previous arterial revascularizations (coronary artery bypass or angioplasty, thromboendarterectomy or stenting of supra-aortic trunks, bypass or angioplasty of lower limb arteries). All these events were confirmed by the available documentation.

Deficits in physical function included poor aerobic capacity, walking problems, falls (at least one fall during the previous 3 years), and deficits in activities of daily living (ADL) (inability to autonomously perform at least one of the six ADLs according to Katz and Akpom [37]).

Subjects’ aerobic capacity was assessed by the step test, whose result has been found to be correlated with maximum oxygen consumption [38]. The time in seconds needed to ascend and descend 2 steps 20 times was measured so that the subject’s aerobic capacity was inversely proportional to this time. A large number of subjects (*N* = 247) were unable to perform the test. These subjects were included in the analysis with the maximum observed time (234 s). Poor aerobic capacity was defined as a step test duration exceeding the median time (≥ 114 s).

Walking problems were defined according to the first item (mobility) of the 5-dimension Euro QoL questionnaire [39], an often used tool for the assessment of quality of life. In particular, any difficulty, even mild, in walking was considered (e.g., when either of the answers “some walking problem” or “extreme walking problem or unable to walk” was provided).

The psychological or mental complaints included poor self-rating of health status, anxiety or depressive symptoms, and cognitive decline as assessed by the Mini Mental State Examination (MMSE) [40].

The self-rating of health status was performed utilizing the visual analog scale attached to the 5-dimension Euro QoL questionnaire [39] on which scores range from 0 (worst perceived health status) to 100 (best perceived health status). Poor self-rating was defined as a score lower than the median value (< 71).

The presence of anxiety or depressive symptoms was defined according to the fifth item of the 5-dimension Euro QoL questionnaire (anxiety/depression) [39] considering any, even if mild, anxious or depressive status.

Cognitive decline was defined as an MMSE score < 24, as suggested in previous studies [41, 42]. This value corresponded to the lower quartile cutoff obtained both in our sample and in an Italian population aged 65–69 [27].

Finally, the following variables were used as adjustment factors: body mass index (BMI, body weight/[standing height]^2^, kg/m^2^) for nutritional status, Physical Activity Scale for the Elderly (PASE) score [43] for reported physical activity and years of education for schooling.

### Statistical analysis

Following the baseline assessment, a second assessment was performed 7 years later. The collection from the Registry Office of mortality data was complete, but only a subgroup of patients returned the new questionnaires and agreed to come to our laboratories; therefore, the number of incident events was insufficient for statistical analysis. Thus, the present analysis is of the cross-sectional type concerning baseline parameters and previous events.

For each of the 11 manifestations of aging, the 5 laboratory variables (proBNPage, ESR, hemoglobin, lymphocytes and creatinine) plus age and sex were compared between subjects with and without the manifestation. Continuous variables are described as the mean ± standard deviation (SD) or as the median and interquartile range in relation to their distribution (Gaussian or non-Gaussian, respectively). The comparisons of such variables between the 2 groups were tested by Student’s *t* test for unpaired data or Mann–Whitney’s *U* test. The comparisons between percentages were assessed by the *χ*^2^ test.

For both univariate and multivariate analyses, only cases without missing values were used. The resulting sample sizes are reported in the tables.

Multivariate analysis was performed by multiple logistic regression with backward elimination of the nonsignificant associations and adjustment for BMI, physical activity (PASE score), and schooling, as these potential confounders may variously affect both the independent and the dependent variables studied. Before the analysis, the 5 laboratory variables that had been found to be strongly correlated with age were tested for multicollinearity together with chronological age by the variance inflation factor (VIF). The analysis produced odds ratios (ORs) with their relative 95% confidence intervals (CIs). Wald statistics are reported as well to allow comparison between the significance levels of the variables, especially when *P* values were equal.

Analyses were performed using SPSS Statistics v. 22 (IBM, Armonk, NY, USA). Two-tailed tests were used throughout, and *P* values < 0.05 were considered significant.

## Results

The main characteristics of our sample are shown in Table 1. The 5 potential markers of aging plus age and sex were variously associated with the 11 age-related manifestations of cardiovascular, physical, and mental impairment. No multicollinearity was found among the laboratory variables plus age after complete VIF analysis: the maximum VIF value was 1.413 (it was associated with hemoglobin when the dependent variable was chronological age), a value below the limit of 3 over which multicollinearity becomes probable. In the following text, the associations found are listed in decreasing order of significance.

**Table 1:** Characteristics of the study sample (*N* = 1117)

Age (years)	73.1 ± 5.6
Male	537 (48.1)
BMI (kg/m^2^)	26.4 ± 4.0
Hypertension	955 (85.5)
Hypercholesterolemia	861 (77.1)
Diabetes	161 (14.4)
Ever smoker	494 (44.2)
Previous myocardial infarction	69 (6.2)
Previous stroke	25 (2.2)
Peripheral artery disease	20 (1.8)
Previous arterial revascularization	66 (5.9)
Heart failure history	12 (1.1)
Atrial fibrillation	25 (2.2)
Step test duration (s)	113 (93–171)
Walking problems	267 (23.9)
Falls	242 (21.7)
ADL deficit	355 (31.8)
Self-rated health (VAS)	70 (55–80)
Anxiety/depression	583 (52.2)
MMSE	27 (24–29)
NT-proBNP (pg/ml)	135 (76–264)
proBNPage (years)	73.7 ± 16.8
ESR (mm/h)	17 (11–30)
Hemoglobin (g/dl)	13.9 ± 1.3
Lymphocytes (%)	29.3 ± 7.5
Creatinine (mg/dl)	0.94 (0.81–1.09)
CRP (mg/dl)	0.19 (0.10–0.40)

Values are the mean ± SD, median (25th–75th percentile) or number (percentage)

*ADL* activities of daily living, *BMI* body mass index, *CRP* C-reactive protein, *ESR* erythrocyte sedimentation rate, *MMSE* Mini Mental State Examination, *VAS* visual analog scale

### Associations with cardiac-cerebral-vascular events

In univariate analysis, age, sex, proBNPage, and creatinine were all strongly associated with previous myocardial infarction. However, in multivariate analysis, only proBNPage and male sex remained associated with this condition (Table 2). The independent associations with previous stroke were less significant (perhaps because of the lower number of cases) and concerned only the percent reduction of lymphocytes and proBNPage. The same two variables were independently associated with peripheral artery disease. Nevertheless, in this case, the association of proBNPage was more significant than that of lymphocytes. Finally, proBNPage had the strongest association with arterial revascularizations, followed by male sex. Creatinine also had a strong univariate association with arterial revascularizations, which was not confirmed in multivariate analysis.

**Table 2:** Univariate and multivariate associations with cardiac-cerebral-vascular events

	Absent	(Number)	Present	(Number)	*P* value	OR (95% CI)	Wald	*P* value*
Previous myocardial infarction								
Age (years)	72.9 ± 5.5	1048	75.7 ± 6.3	69	< 0.0001	-	-	-
Sex (male)	487 (46.5)	1048	50 (72.5)	69	< 0.0001	5.03 (2.59–9.77)	22.7	< 0.0001
proBNPage (years)	72.4 ± 15.9	1026	93.2 ± 17.4	67	< 0.0001	1.08 (1.07–1.10)	79.0	< 0.0001
ESR (mm/h)	17 (11–30)	1039	21.5 (9.5–35)	68	0.41	-	-	-
Hemoglobin (g/dl)	13.9 ± 1.3	1042	13.7 ± 1.5	69	0.08	-	-	-
Lymphocytes (%)	29.2 (24.6–33.6)	1039	28.3 (20.6–34.3)	69	0.17	-	-	-
Creatinine (mg/dl)	0.93 (0.81–1.08)	1042	1.15 (0.94–1.30)	69	< 0.0001	-	-	-
Previous stroke								
Age (years)	73.0 ± 5.6	1092	75.8 ± 6.3	25	0.01	-	-	-
Sex (male)	525 (48.1)	1092	12 (48.0)	25	0.99	-	-	-
proBNPage (years)	73.4 ± 16.8	1070	83.8 ± 15.2	23	0.003	1.02 (1.00–1.05)	3.7	0.05
ESR (mm/h)	17 (11–30)	1082	26 (12–39)	26	0.18	-	-	-
Hemoglobin (g/dl)	13.9 ± 1.3	1086	13.8 ± 1.2	25	0.59	-	-	-
Lymphocytes (%)	29.2 (24.5–33.7)	1083	25.9 (20.9–29.6)	25	0.007	0.92 (0.87–0.98)	7.0	0.008
Creatinine (mg/dl)	0.94 (0.81.1.08)	1087	1.05 (0.88–1.21)	24	0.02	-	-	-
Peripheral artery disease								
Age (years)	73.0 ± 5.6	1097	75.2 ± 5.6	20	0.09	-	-	-
Sex (male)	524 (47.8)	1097	13 (65.0)	20	0.13	-	-	-
proBNPage (years)	73.4 ± 16.6	1073	87.2 ± 20.3	20	0.0003	1.04 (1.01–1.06)	7.9	0.005
ESR (mm/h)	17 (11–30)	1087	19 (6–26)	20	0.41	-	-	-
Hemoglobin (g/dl)	13.9 ± 1.3	1091	13.9 ± 1.7	20	0.97	-	-	-
Lymphocytes (%)	29.1 (24.6–33.7)	1088	23.3 (19.5–31.4)	20	0.01	0.94 (0.88–0.99)	4.1	0.04
Creatinine (mg/dl)	0.94 (0.81–1.08)	1091	1.11 (0.94–1.17)	20	0.03	-	-	-
Previous arterial revascularization								
Age (years)	73.0 ± 5.7	1051	74.6	66	0.03	-	-	-
Sex (male)	483 (46.0)	1051	54 (81.8)	66	< 0.0001	7.40 (3.58–15.31)	29.1	< 0.0001
proBNPage (years)	72.9 ± 16.7	1030	86.7 ± 14.9	63	< 0.0001	1.06 (1.04–1.08)	44.3	< 0.0001
ESR (mm/h)	17 (11–30)	1043	17.5 (13–29.5)	64	0.67	-	-	-
Hemoglobin (g/dl)	13.9 ± 1.3	1045	13.9 ± 1.5	66	0.68	-	-	-
Lymphocytes (%)	29.2 (24.6–33.7)	1042	27.8 (20.0–31.8)	66	0.02	-	-	-
Creatinine (mg/dl)	0.93 (0.81–1.08)	1046	1.08 (0.92–1.22)	65	< 0.0001	-	-	-

Values for absent and present are mean ± SD, median (25th–75th percentile) or number (percentage)

Multivariate analysis (*N* = 1083) adjusted for BMI and PASE score

*BMI* body mass index, *CI* confidence interval, *ESR* erythrocyte sedimentation rate, *OR* odds ratio, *PASE* Physical Activity Scale for the Elderly

^*^Multivariate *P* values refer to residual significant associations after multiple logistic regression with the backward elimination procedure

A sensitivity analysis performed after the exclusion of the subjects with atrial fibrillation or heart failure showed similar associations: male sex (OR 5.42, 95% CI 2.66–11.07) and proBNPage (OR 1.09, 95% CI 1.07–1.11) with previous myocardial infarction; proBNPage (OR 1.07, 95% CI 1.05–1.09) but not lymphocytes with previous stroke; proBNPage (OR 1.05, 95% CI 1.02–1.07) but not lymphocytes with peripheral artery disease; and male sex (OR 9.72, 95% CI 4.35–21.70) and proBNPage (OR1.07, 95% CI 1.05–1.10) with previous arterial revascularizations.

### Associations with manifestations of physical dysfunction

Four variables were independently associated with poor aerobic capacity: chronological age, proBNPage, female sex, and ESR (low values of hemoglobin and lymphocytes had strong univariate associations with poor aerobic capacity, but their associations were not confirmed in multivariate analysis) (Table 3). The same 4 variables plus lymphocytes were independently associated with walking problems, whereas the univariate association of hemoglobin was again not confirmed by multivariate analysis. Only female sex and chronological age were independently associated with falls, whereas low hemoglobin, creatinine, and proBNPage (borderline significance) were associated with falls only in univariate analysis. Similarly, only female sex and (at a lower level of significance) chronological age were independently associated with deficits of at least one ADL. Other nonindependent associations with ADL deficits concerned ESR and low hemoglobin.

**Table 3:** Univariate and multivariate associations with deficits of physical function

	Absent	(Number)	Present	(Number)	*P* value	OR (95% CI)	Wald	*P* value*
Poor aerobic capacity (step test duration ≥ 114 s)								
Age (years)	71.3 ± 4.4	568	74.9 ± 6.1	549	< 0.0001	1.14 (1.11–1.17)	78.1	< 0.0001
Sex (male)	322 (56.7)	568	215 (39.2)	549	< 0.0001	0.46 (0.35–0.60)	30.7	< 0.0001
proBNPage (years)	69.4 ± 14.6	562	78.2 ± 17.7	895	< 0.0001	1.03 (1.02–1.04)	32.5	< 0.0001
ESR (mm/h)	16 (10–26)	564	21 (13–34)	543	< 0.0001	1.01 (1.00–1.02)	6.3	0.01
Hemoglobin (g/dl)	14.1 ± 1.2	565	13.7 ± 1.3	546	< 0.0001	-	-	-
Lymphocytes (%)	29.9 (25.7–34.2)	564	28.2 (23.5–32.9)	544	0.0001	-	-	-
Creatinine (mg/dl)	0.94 (0.81–1.09)	567	0.94 (0.82–1.09)	544	0.50	-	-	-
Walking problems								
Age (years)	72.4 ± 5.3	850	75.1 ± 6.2	267	< 0.0001	1.08 (1.05–1.12)	30.3	< 0.0001
Sex (male)	435 (51.2)	850	102 (38.2)	267	0.0002	0.54 (0.40–0.74)	14.9	0.0001
proBNPage (years)	72.0 ± 16.0	835	78.9 ± 18.1	258	< 0.0001	1.02 (1.01–1.03)	12.1	0.0005
ESR (mm/h)	16 (10–29)	843	23 (13.5–34.5)	264	< 0.0001	1.01 (1.00–1.02)	6.0	0.01
Hemoglobin (g/dl)	14.0 ± 1.3	845	13.6 ± 1.3	266	< 0.0001	-	-	-
Lymphocytes (%)	29.3 (24.8–33.8)	843	28.2 (23.9–32.8)	265	0.03	0.98 (0.96–1.00)	4.3	0.04
Creatinine (mg/dl)	0.94 (0.82–1.09)	846	0.93 (0.79–1.11)	265	0.62	-	-	-
Falls								
Age (years)	72.7 ± 5.5	875	74.5 ± 5.9	242	< 0.0001	1.07 (1.04–1.09)	22.6	< 0.0001
Sex (male)	459 (52.5)	875	78 (32.2)	242	< 0.0001	0.43 (0.32–0.59)	28.3	< 0.0001
proBNPage (years)	73.1 ± 16.3	857	75.7 ± 18.5	236	0.052	-	-	-
ESR (mm/h)	17 (11–30)	866	19 (12–30)	241	0.09	-	-	-
Hemoglobin (g/dl)	14.0 ± 1.3	870	13.7 ± 1.3	241	0.0002	-	-	-
Lymphocytes (%)	29.3 (24.5–33.9)	868	28.6 (24.3–32.5)	240	0.12	-	-	-
Creatinine (mg/dl)	0.95 (0.82–1.09)	871	0.91 (0.78–1.07)	240	0.009	-	-	-
ADL deficit								
Age (years)	72.8 ± 5.4	760	73.7 ± 6.1	355	0.01	1.04 (1.01–1.06)	8.5	0.004
Sex (male)	404 (53.2)	760	133 (37.5)	355	< 0.0001	0.49 (0.38–0.64)	27.3	< 0.0001
proBNPage (years)	73.5 ± 16.1	746	74.0 ± 18.3	345	0.70	-	-	-
ESR (mm/h)	16 (10–29)	755	21 (13–33)	350	0.0001	-	-	-
Hemoglobin (g/dl)	14.0 ± 1.3	756	13.8 ± 1.3	353	0.004	-	-	-
Lymphocytes (%)	29.0 (24.5–33.6)	754	29.2 (24.4–33.6)	352	0.92	-	-	-
Creatinine (mg/dl)	0.94 (0.82–1.09)	756	0.94 (0.80–1.09)	353	0.63	-	-	-

Values for absent and present are mean ± SD, median (25th–75th percentile) or number (percentage)

Multivariate analysis (*N* = 1083) adjusted for BMI

*ADL* activities of daily living, *BMI* body mass index, *CI* confidence interval, *ESR* erythrocyte sedimentation rate, *OR* odds ratio

^*^Multivariate *P* values refer to residual significant associations after multiple logistic regression with the backward elimination procedure

After exclusion of the subjects with a history of atrial fibrillation or heart failure, similar associations were found: age (OR 1.13, 95% CI 1.10–1.13), sex (OR 0.45, 95% CI 0.34–0.60), proBNPage (OR 1.03, 95% CI 1.02–1.04), and ESR (OR 1.01, 95% CI 1.0–1.02) with poor aerobic capacity; age (1.07, 95% CI 1.04–1.10), sex (OR 0.46, 95% CI 0.34–0.64), proBNPage (OR 1.02, 95% CI 1.01–1.03), lymphocytes (OR 0.97, 95% CI 0.95–0.99) but not ESR with walking problems; age (OR 1.07, 95% CI 1.04–1.10) and sex (OR 0.42, 95% CI 0.31–0.57) with falls; and age (OR 1.03, 95% CI 1.00–1.05) and sex (OR 0.45, 95% CI 0.34–0.59) with ADL deficits.

### Associations with psychological or mental complaints

ProBNPage, female sex, ESR, and chronological age were independently associated with poor self-rated health, whereas low values of hemoglobin and lymphocytes were associated only in univariate analysis (Table 4). Only female sex was associated with an anxious and/or depressive status. Other nonindependent associations concerned ESR, low hemoglobin, age, and proBNPage. Finally, only chronological age was independently associated with cognitive decline (MMSE < 24), which was also nonindependently associated with proBNPage, female sex, low hemoglobin, and ESR.

**Table 4:** Univariate and multivariate associations with psychological or mental complaints

	Absent	(Number)	Present	(Number)	*P* value	OR (95% CI)	Wald	*P* value*
Poor self-rated health (visual analog scale < 71)								
Age (years)	72.0 ± 5.1	476	73.9 ± 5.8	628	< 0.0001	1.03 (1.00–1.06)	4.9	0.03
Sex (male)	261 (54.8)	476	272 (43.3)	628	0.0001	0.65 (0.50–0.85)	10.1	0.002
proBNPage (years)	70.0 ± 15.1	469	76.1 ± 17.7	612	< 0.0001	1.02 (1.01–1.02)	13.6	0.0002
ESR (mm/h)	16 (9–26)	472	21 (12–32)	622	< 0.0001	1.01 (1.00–1.02)	5.4	0.02
Hemoglobin (g/dl)	14.1 ± 1.2	473	13.8 ± 1.3	625	0.0008	-	-	-
Lymphocytes (%)	29.7 (25.4–34.1)	472	28.7 (24.1–33.3)	623	0.01	-	-	-
Creatinine (md/dl)	0.94 (0.82–1.08)	473	0.94 (0.81–1.10)	625	0.78	-	-	-
Anxiety/depression								
Age (years)	72.9 ± 5.5	534	73.4 ± 5.7	583	0.02	-	-	-
Sex (male)	310 (58.1)	534	227 (38.9)	583	< 0.0001	0.47 (0.37–0.61)	34.4	< 0.0001
proBNPage (years)	72.6 ± 15.8	520	74.6 ± 17.6	573	0.048	-	-	-
ESR (mm/h)	16 (10–27)	526	20 (12–31)	581	0.0001	-	-	-
Hemoglobin (g/dl)	14.1 ± 1.3	530	13.8 ± 1.3	581	0.0001	-	-	-
Lymphocytes (%)	29.4 (25.1–33.8)	529	28.7 (24.2–33.5)	579	0.11	-	-	-
Creatinine (md/dl)	0.96 (0.83–1.10)	528	0.92 (0.80–1.08)	583	0.03	-		-
MMSE score < 24								
Age (years)	72.5 ± 5.3	890	75.2 ± 6.3	227	< 0.0001	1.05 (1.02–1.08)	10.2	0.001
Sex (male)	452 (50.8)	890	85 (37.4)	227	0.0003	-	-	-
proBNPage (years)	72.8 ± 16.4	875	77.3 ± 17.9	218	0.0003	-	-	-
ESR (mm/h)	17 (11–30)	883	22 (11–34)	224	0.009	-	-	-
Hemoglobin (g/dl)	14.0 ± 1.3	885	13.7 ± 1.3	226	0.0006	-	-	-
Lymphocytes (%)	29.1 (24.5–33.7)	884	28.8 (24.4–33.5)	224	0.49	-	-	-
Creatinine (md/dl)	0.94 (0.81–1.10)	886	0.93 (0.80–1.05)	225	0.17	-	-	-

Values for absent and present are mean ± SD, median (25th–75th percentile) or number (percentage)

Multivariate analysis (*N* = 1083) adjusted for BMI, PASE score, and schooling

*BMI* body mass index, *CI* confidence interval, *ESR* erythrocyte sedimentation rate, *MMSE* Mini Mental State Examination, *OR* odds ratio, *PASE* Physical Activity Scale for the Elderly

^*^Multivariate *P* values refer to residual significant associations after multiple logistic regression with the backward elimination procedure

After exclusion of the subjects with a history of atrial fibrillation or heart failure, similar associations were found: age (OR 1.04, 95% CI 1.01–1.07), sex (OR 0.59, 95% CI 0.46–0.77), proBNPage (OR 1.01, 95% 1.00–1.02), and ESR (OR 1.01, 95% CI 1.00–1.02) with poor self-rated health; sex only (OR 0.44, 95% CI 0.34–0.56) with anxiety/depression; and age (OR 1.08, 95% CI 1.5–1.11) plus sex (0.63, 95% CI 0.46–0.87) with MMSE score < 24.

Table 5 summarizes the overall performance of proBNPage as an indicator of aging compared to chronological age. Associations with all-cause and cardiovascular mortality have been obtained in previous studies on the same population [16, 23], whereas the other associations are from the present study. Overall, the table considers 13 manifestations of aging. ProBNPage was independently associated with 9 manifestations, and with 7 of them, the association was more significant than that of chronological age.

**Table 5:** Associations of proBNPage with some manifestations of aging: comparison with chronological age

Independent associations more significant than those with chronological age	Independent associations less significant than those with chronological age	Univariate-only associations	No association
All-cause mortality	Poor aerobic capacity	MMSE score < 24	ADL deficit
Cardiovascular mortality	Walking problems	Anxiety/depression	
Previous myocardial infarction		Falls	
Previous stroke			
Peripheral artery disease			
Arterial revascularizations			
Poor self-rated health			

## Discussion

In the present study, we aimed to test the associations between proBNPage (a recently proposed indicator of biological age) and age-related changes in various biological and psychological functions in comparison with other known biomarkers. We found that proBNPage was independently associated with 7 of 11 cardiovascular, physical, and mental manifestations of aging, and that for 5 of them, the association was more significant than that of chronological age. In particular, proBNPage more strongly predicted the cardiovascular manifestations of aging but was also associated independently or nonindependently with some typical physical and psychological complaints of advanced age. None of the other laboratory variables that had highly significant associations with both chronological age and all-cause mortality provided comparable performances.

### Cardiac-cerebral-vascular events

In developed countries, cardiovascular diseases are the first cause of death [44] and probably also of aging; therefore, one could say that the age of an individual is the age of his/her arteries [45]. Previously, we had already shown that among a series of aging-associated variables, NTproBNP and proBNPage were the best indicators of cardiovascular [23] and, consequently, all-cause [16] mortality. These associations have also been reported in other studies [21, 46]. In the present study, proBNPage was the parameter most associated with previous myocardial infarction, peripheral artery disease, and arterial revascularizations. In addition, proBNPage remained independently associated even with previous stroke, although with a lower significance than low lymphocytes. Even though noncardiac sites of NTproBNP release may exist [21], the main site is represented by ventricular and atrial cardiomyocytes undergoing mechanical or ischemic damage [21, 46–48]. In the presence of generalized atherosclerotic disease, even with prevalent peripheral localization, some degree of coronary involvement is easily associated, and, with an ischemic mechanism, this could favor the release of NTproBNP.

Among the laboratory variables considered here, only low lymphocytes remained associated, independent of proBNPage, with previous stroke and peripheral artery disease. In at least another study [6], low lymphocytes were found to be associated with an increase in all-cause mortality, and in the present study, we have evidenced that this, in particular, might be related to atherosclerotic disease. This is also in agreement with the data of Horne et al. [49], who showed that the inflammatory status associated with cardiovascular risk can be more accurately predicted with the count of leukocyte subpopulations, rather than with leukocyte count. In particular, these authors showed a protective role of lymphocytes that is not easily explainable. More recently, the neutrophil/lymphocyte ratio has emerged as a relevant indicator of atherosclerosis [50, 51] in all main vascular districts [52–54].

Creatinine was associated with all cardiovascular events and, particularly, with previous myocardial infarction and arterial revascularizations, but in univariate analysis only.

Finally, male sex was independently associated with previous myocardial infarction and peripheral artery disease, whereas chronological age was not associated with any cardiac-cerebral-vascular event in the multivariate analyses that included proBNPage.

### Deficits of physical function

Age and female sex were independently associated with high significance with all physical deficits. However, proBNPage was also associated, with high significance and independent of age and sex, with poor aerobic capacity and walking problems and had a borderline univariate association with falls. This is in agreement with previous studies that have shown that B-type natriuretic peptide levels are increased in frail older adults and are inversely associated with walking capacity and grip strength [43, 55]. These relationships might be explained, given that poor aerobic capacity, walking problems, and possible associated falls may be manifestations of physical frailty due to impaired cardiovascular function [43].

In this study, proBNPage had no relationship with ADL deficits (but no laboratory variable was associated with ADL deficits independent of age and sex).

Like proBNPage, ESR proved to be predictive of poor aerobic capacity and walking problems. This may be explained at least in part by the fact that mobility is also influenced by osteo-articular inflammatory pathologies.

### Psychological or mental complaints

In this group, only female sex was independently associated with anxiety/depression, and only age was independently associated with cognitive decline; both were associated with poor self-rated health.

Nevertheless, proBNPage was the variable most significantly associated independent of age and sex with poor self-rated health and was also associated only in univariate analysis with cognitive decline and anxiety/depression, confirming previous studies on this subject [31–34].

The strong relationship between proBNPage and cardiovascular pathologies leads to the conclusion that the presence of these pathologies may also have a strong influence on the self-rating of health status. With the intermediation of endothelial dysfunction, hypertension is a relevant cause of cardiovascular damage, and both hypertension and endothelial dysfunction have been found to be associated with cognitive impairment [56, 57]. The impact on cognitive aging of even subtle alterations of cardiovascular hemodynamics, including both cardiac output and arterial stiffening, has recently been reviewed [58]. Thus, as an important marker of cardiovascular damage, NT-proBNP might also be indirectly associated with some manifestations of cognitive impairment. Alternatively, the only univariate associations of proBNPage with cognitive decline and mood status might be a consequence of the strong associations of proBNPage with chronological age [16], which in turn is independently associated with those 2 conditions. In addition, proBNPage is also independently associated with step test duration, and the latter has been found to be strongly associated with cognitive deficit and decline [59], reflecting the known relationship between physical fitness and mental health [60].

Among the other laboratory variables, only ESR was independently associated with poor self-rating of health status, perhaps again reflecting the influence that inflammatory conditions and osteo-articular pain may have on the perception of well-being in older adults. Only in univariate analysis was low hemoglobin associated with all manifestations of psychological disturbance, probably because of its relationship with age and female sex.

### Is proBNPage a true marker of biological age or just a marker of cardiovascular damage?

NT-proBNP is mainly released by myocardial tissue stressed by mechanical or ischemic causes [47, 61], so it behaves quite well as a marker of cardiovascular damage. In fact, as expected, the highest relative risks detected in this study concerned the associations of proBNPage with myocardial infarction (+ 8% for each year of proBNPage), arterial revascularizations (+ 6%), and peripheral artery disease (+ 4%), whereas the associations with noncardiovascular manifestations (walking problems, poor self-rated health, poor aerobic capacity) were weaker (+ 2–3%). Additionally, they might also have been influenced by cardiovascular function. Despite that, we believe that proBNPage should not be considered a false indicator of biological age for the following reasons. (1) The impairment of cardiac and circulatory function is both an important consequence and a cause of the aging process [58, 62–64]. (2) Among all biochemical parameters, NT-proBNP is probably the one most correlated with chronological age [12, 16]. (3) An increase in NT-proBNP is also associated with pathologies that are apparently far from cardiovascular function, such as liver cirrhosis [65], chronic obstructive pulmonary disease [67], and anemia [35]. (4) In a recent study by Gomez-Cabrero et al. [67], among more than 35,000 clinical and laboratory biomarkers obtained from genomic, proteomic, and metabolomic data in four population-based European cohorts, NT-proBNP was the marker most strongly associated with frailty (as defined by Fried et al. [68]) in the subjects without disability. (5) In an older population, another recent study [69] showed that NT-proBNP was the only independent risk factor for decreased intrinsic capacity among 20 laboratory variables, being associated with abnormal locomotion, hearing, vision, and psychological domains.

Nevertheless, any manifestation of aging, including frailty, might be connected more or less directly with some abnormality in the cardiocirculatory system; however, for this reason, if a single laboratory biomarker of aging has to be chosen, the choice of NT-proBNP would probably be the best.

### Limitations

All the examined associations were of the cross-sectional type. Unfortunately, relatively few subjects participated in the longitudinal study, so the number of events during the follow-up period, except deaths, was too small to allow an adequate statistical assessment. Information concerning some aging-associated pathologies, such as cancer, was not available, as it did not concern the original objective of the study. Some definitions of aging manifestations were based on data obtained from self-reported questionnaires, which may capture a different spectrum than performance-based tests. In addition, the possibility that other laboratory variables may have better performance than the variables considered here cannot be excluded. Finally, other studies are needed to compare the performance of proBNPage with that of other markers of biological age, such as telomere length [10] and epigenetic clock [1, 2] and to assess the possibility of reducing proBNPage with antiaging treatments.

## Conclusions

Keeping in mind the above limitations, in this study, proBNPage was found to be independently associated with previous myocardial infarction, previous stroke, peripheral artery disease, arterial revascularizations, poor self-rated health, poor aerobic capacity, and walking problems, and to be nonindependently associated with cognitive decline, anxiety/depression, and falls. Regarding the confounding possibly caused by the common relationship with chronological age, for 7 manifestations, the associations with proBNPage were independent of chronological age, and for 5 of them (all the cardiac-cerebral-vascular manifestations plus poor self-rated health), the association with proBNPage was stronger than that with age. However, despite these associations with some manifestations of aging (in addition to the previously shown associations with all-cause mortality and disease count), it is not probable that NTproBNP plays any causal role in the aging process. Most likely, this peptide is only a marker of processes and events that have different causes. Nevertheless, this should not prevent the utilization of proBNPage as a surrogate endpoint in the assessment of anti-aging treatments, thus avoiding the need for long longitudinal investigations. This matter will be the subject of further studies.

## Data Availability

The data that support the findings of this study are available from the corresponding author upon reasonable request.
